# Effect of the COVID-19 Pandemic Preparation and Response on Essential Health Services in Primary and Tertiary Healthcare Settings of Amhara Region, Ethiopia

**DOI:** 10.4269/ajtmh.21-0354

**Published:** 2021-09-20

**Authors:** Wendemagegn Enbiale, Seid Getahun Abdela, Meaza Seyum, Dereje Bedanie Hundie, Kassawmar Angaw Bogale, Koku Sisay Tamirat, Mulat Birhanu Feleke, Muluken Azage, Dabere Nigatu, Henry J. C. de Vries

**Affiliations:** ^1^ College of Medicine and Health Sciences, Bahir Dar University, Bahir Dar, Ethiopia;; ^2^ Amsterdam UMC, University of Amsterdam, Department of Dermatology, Amsterdam Institute for Infection and Immunity (AI&I), Location Academic Medical Centre, Amsterdam, The Netherlands;; ^3^ Department of Internal Medicine, College of Medicine and Health Sciences, Wollo University, Dessie, Ethiopia;; ^4^ Addis Alem Hospital, Bahir Dar, Ethiopia;; ^5^ Department of Epidemiology and Biostatistics, Institute of Public Health, College of Medicine and Health Sciences, University of Gondar, Gondar, Ethiopia;; ^6^ WagHimra Zone Health Department, Sekota, Ethiopia

## Abstract

Countries like Ethiopia have had to make difficult decisions to balance between the demands of the COVID-19 pandemic and maintaining the essential health service delivery. We assessed the effect of preventive COVID-19 measures on essential healthcare services in selected health facilities of Ethiopia. In a comparative cross-sectional study, we analyzed and compared data from seven health facilities over two periods: the pre-COVID-19 period before the first reported COVID-19 case in the country and during the COVID-19 period. Data were summarized using descriptive statistics and the independent *t* test. During the COVID-19 period the average number of monthly patient visits in the emergency department, pediatrics outpatient, and adult outpatient dropped by 27%, 30%, and 27%, respectively compared with the pre-COVID-19 period. Family planning; institutional delivery; childhood immunization; antenatal care-, hypertension- and diabetic patient follow-up, did not vary significantly between pre-COVID-19 and during COVID-19. Moreover, the monthly average number of tuberculosis (TB) and HIV patients who visited health facilities for drug refill and clinical evaluation did not vary significantly during the two periods. In conclusion, the study highlights that the effect of public restrictions to mitigate the COVID-19 pandemic on essential care systems should be considered.

## INTRODUCTION

Sever acute respiratory syndrome coronavirus 2 (SARS-CoV-2) that causes coronavirus disease 2019 (COVID-19) has spread rapidly since the end of 2019, to become a pandemic and a public health emergency of international concern.^1^^,^^2^ Countries around the world maximized their efforts to prevent the transmission of the virus and provide care for COVID-19 patients. However, the main challenge was to meet the demand imposed by the increasing number of COVID-19 patients, while maintaining the delivery of essential health services.^3^^,^^4^ A well-organized and prepared health system has the capacity to maintain equitable access to essential service delivery throughout an emergency period, limiting direct and indirect morbidity and mortality.^4^ Maintaining community trust in the essential health system capacity and safety in a time of emergency is key to ensure appropriate healthcare-seeking behavior and adherence to public health advice.^5^

When health systems are overwhelmed, both direct mortality from the pandemic and indirect mortality from preventable and treatable conditions increase substantially. Analyses from the 2014–2015 Ebola outbreak suggest that more deaths caused by malaria, measles, tuberculosis (TB), HIV/AIDS and health system failures outweighed deaths from Ebola.^6^^–^^8^ In May 2020, WHO surveyed the impact of COVID-19 on health services for noncommunicable diseases in 155 countries. Although the survey confirmed that the impact is global, low-income countries in particular proved to be the most affected.^9^

Countries like Ethiopia will need to make difficult decisions to balance the demands from the COVID-19 pandemic, while simultaneously engaging in strategic planning and coordinated action to maintain essential health service delivery, mitigating the risk of system collapse.^4^

The WHO prepared a guideline on how to continue essential services during the COVID-19 pandemic. The guideline recommends continuing vaccinations, chronic diseases follow-up, maternal and child healthcare, by taking in to consideration the extent of the pandemic in each locality. In areas with a relatively limited number of COVID-19 cases, the health system may have the capacity to maintain routine service delivery in addition to managing COVID-19 cases. But in case of increased caseload, and/or compromization of the health workforce, strategic shifts are required to ensure that increasingly limited resources provide maximum benefit for a population.^10^^,^^11^

Ethiopia, a country with 1.5% of the global population (*N* = 117,557,128), reported the first COVID-19 case on March 13, 2020. As of May 23, 2021 Ethiopia had registered only 0.16% of the global number of COVID-19 cases and 0.12% of the global deaths from the disease.^12^ Following the WHO advice after confirming the first case, the Ethiopian federal ministry of health (MOH) initiated different prevention strategies such as a partial and selective lockdown, the prohibition of mass gatherings such as mass praying, and the closure of schools. At the same time, the MOH ensured the continuation of essential services, such as reproductive, maternal, neonatal, child, and adolescent health services, and the management of major communicable and noncommunicable diseases, surgical care, and emergency and critical care.^13^ A study in Ethiopia to study the effect of COVID-19 on essential health services over 8 weeks (4 weeks before and 4 weeks after the implementation of preventive measures) revealed that the case flow for almost all the services has declined.^14^^,^^15^ However, the results may not be generalizable since the study was limited to one referral hospital and had a short duration. A PubMed search revealed some publications on the effect of COVID-19 on specific health services (cancer, mental health, radiology service, and noncommunicable diseases),^16^^–^^18^ but the effect of COVID-19 on essential healthcare was not assessed. Therefore, here we study the effect of preventive COVID-19 measures on essential healthcare services in selected primary and tertiary care settings of Amhara region, Ethiopia. Such information may guide policy decisions and balance the demands imposed by the COVID-19 pandemic, while maintaining essential health services. This will also help in mitigation of the risk of health system collapse.

## MATERIALS AND METHODS

### Study design.

A comparative cross-sectional study to assess essential services uptake before and during the COVID-19 pandemic. We used monthly aggregated data from July 7, 2019 to July 6, 2020 (one Ethiopian fiscal year).

### Study setting and period.

Amhara, the second most populous Regional State in the nation, is one of the 10 administrative regions in Ethiopia with an estimated 30 million inhabitants.^19^ Based on the report of the MOH, more than 90% of the population in the region has access to health facility. The three-tier healthcare system ranges from primary healthcare units to higher tertiary level specialized hospitals. Primary healthcare units (PHCU) comprise health posts, health centers, and primary hospitals, serving 4,000 to 100,000 inhabitants. Secondary healthcare comprises general hospitals covering a population of 1–1.5 million people, and the tertiary care system comprise referral and specialized hospitals covering a population of 3.5–5 million people. All three healthcare systems provide essential healthcare services.^20^ In the Amhara region, there are 98 hospitals and 825 health centers providing essential health services. In this study, we included data from four hospitals and three PHCUs (Table 1).

**Table 1:** Characteristics of health facilities in Amhara region included in the study

Name of health facility	Catchment area population	Number of health professionals
Azezo health center	69,628	Nurses = 15, Midwives = 4, Pharmacists = 13, Health officer = 4, and Laboratory technologists and technicians = 4
Adisalem Hospital	300,000	General practitioners = 19, Specialists = 4, Nurses = 38, Midwives = 16, Pharmacists = 10 and Laboratory technologists and technicians = 10
BahirDar health center	76,343	General practitioner = 1, Nurses = 18, health officers = 8, Midwives = 7, Pharmacy = 4 and Laboratory technologists and technicians = 5
Haik health center	71,829	Nurses = 13, Midwives = 6, Health officers, Pharmacists, Laboratory technologists, and technicians = 5
Tefera Hailu General hospital	Above 1 million	General practitioners = 17, Specialists = 4, Nurses = 93, Midwives = 24, and Laboratory technologists and technicians = 12
Dessie comprehensive specialized hospital	Above 8 million	General practitioners = 85, Specialists = 42, Nurses = 210, Midwives = 47, Pharmacists = 48, and Laboratory technologists and technicians = 38
Tibebe Ghion Teaching Hospital	Above 5 million	General practitioners = 16, Specialists = 123, Nurses = 284, Midwives = 69, Pharmacists = 42, and Laboratory technologists and technicians = 48

These health facilities were selected after considering their location and convenience.

### Study population.

Medical records of the visitors of the abovementioned health facilities from July 7, 2019 to July 6, 2020.

### Data collection and sources of data.

Data were retrieved from Health Management Information Systems (HMIS). The HMIS data from two periods were analyzed and compared: the pre-COVID-19 period from July 7, 2019 to March 8, 2020 (before the first reported COVID-19 case in Ethiopia); and the during COVID-19 period from March 9, to July 6, 2020 (after the first reported case in Ethiopia). We collected the following data: name and tier of visited healthcare facility, number of patients visits to different essential health services (such as number of patients who visited adult and pediatric emergency; outpatient surgical, medical, and pediatrics departments; the number of newly diagnosed HIV cases; number of visit to antiretroviral therapy (ART) clinic), and maternal and child care indicators (number of deliveries stratified by route, vaccinations, if applicable).

### Data management.

Data at district level from health facility and regional registers were entered in Microsoft Excel by two independent persons and cross-checked by the principal investigator. Data were imported in SPSS (Version 20) (Chicago, Illinois) for data quality assessment and analysis. The data were presented using descriptive statistics: frequency and measures of central tendency. The Independent *t* test was used to compare means before and during the COVID-19 periods.

## RESULTS

### Adult medical and surgical emergency department patient flow.

**Figure 1.**
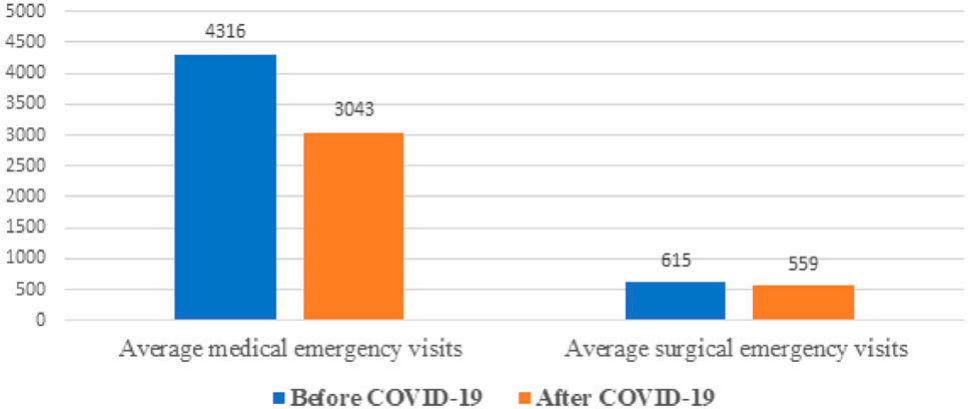
Monthly medical and surgical emergency visits pre-COVID-19, and during the COVID-19 pandemic from selected health facilities in Amhara region, Ethiopia, from July 7, 2019 to March 8, 2020, and from March 9, 2020 to July 6, 2020. This figure appears in color at www.ajtmh.org.

The average number of monthly patients in the emergency department in the pre-COVID-19 period was 4,932. During COVID-19, it dropped by 27% to 3,603 patients, and the lowest number of patients was documented in April 2020 (2,889), yet there was no significant difference in the number of emergency visits between the pre-COVID-19 and during COVID-19 periods (*P* = 0.3) (Figure 1).The emergency visit at tertiary level hospitals significantly lower during COVID-19 (*P* = 0.009) whereas the primary healthcare level emergency visit didn’t vary (*P* = 0.08).

### Outpatient patient flow.

**Figure 2.**
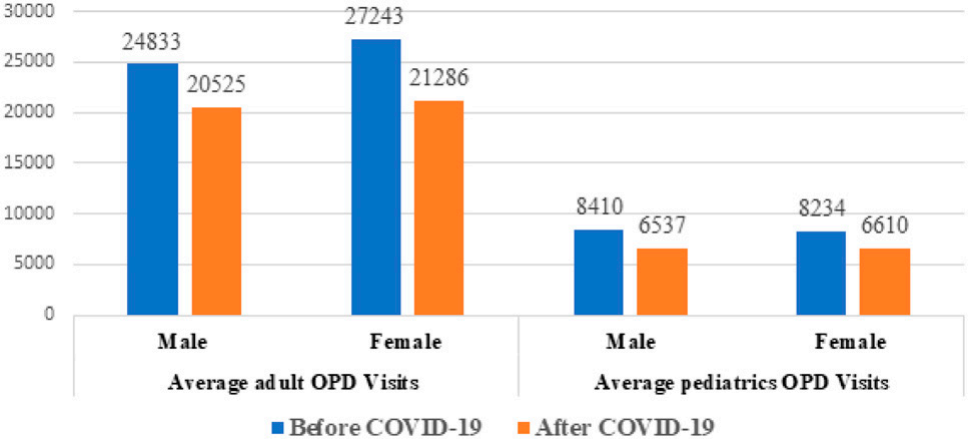
Monthly adult and pediatric outpatient department visitors pre-COVID-19, and during the COVID-19 pandemic from selected health facilities in Amhara region, Ethiopia, from July 7, 2019 to March 8, 2020, and from March 9, 2020 to July 6, 2020. This figure appears in color at www.ajtmh.org.

Overall, 185,746 children visited the pediatrics outpatient department (OPD). The number of children who visited the OPD during the pre-COVID-19 was 16,602. This was significantly higher than the number of children (12,106) who visited the OPD during the COVID-19 period, (*P* = 0.005). The number of pediatric outpatients visit at primary healthcare facility significantly decreased during COVID-19 (*P* = 0.04). The tertiary level hospitals’ pediatric outpatients visit didn’t vary significantly (*P* = 0.3). Similarly, more than half a million (583,859) adult patients visited the outpatient departments overall. The average monthly number of adults who visited the OPD during the pre-COVID-19 period was 52,165. This number was significantly higher than the number of adults (38, 120) who visited the OPD during the COVID-19 period (Figure 2) (*P* = 0.014). The number of adult OPD visit at primary healthcare facility didn’t vary significantly pre-COVID-19 and during COVID-19 period (*P* = 0.2). The tertiary level hospitals adult OPD visit during the COVID-19 period was significantly lower than the pre-COVID-19 period (*P* = 0.02).

### Maternal, child health, and family planning visits.

**Figure 3.**
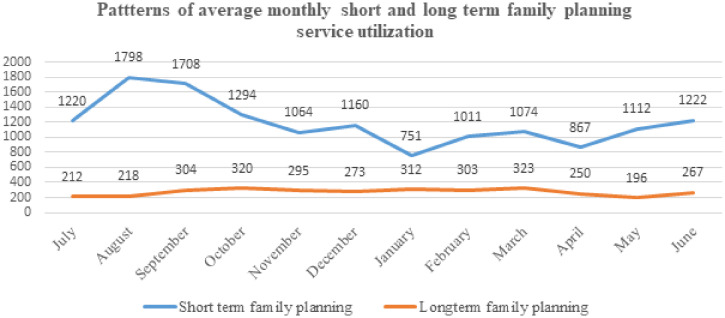
Short- and long-term family planning uptakes in pre-COVID-19, and during the COVID-19 pandemic from selected health facilities in Amhara region, Ethiopia, from July 7, 2019 to March 8, 2020, and from March 9, 2020 to July 6, 2020. This figure appears in color at www.ajtmh.org.

In the pre COVID-19 periods, the monthly average visits for short-term family planning was 1,530 and dropped by 14% to 1,328 during the COIVD-19 period. Yet, the monthly average long-term family planning utilization was 256 and 259, respectively (Figure 3). Both, short and long-term family planning did not vary significantly between pre-COVID-19 and during COVID-19.

**Figure 4.**
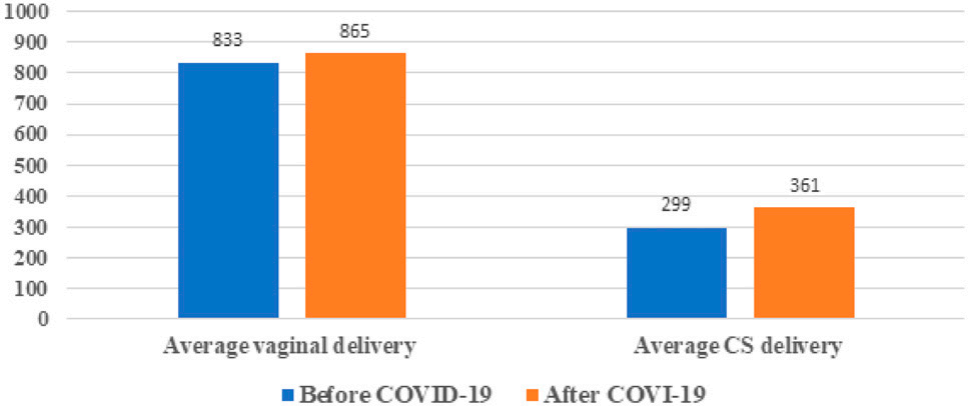
Average monthly vaginal and CS delivery pre-COVID-19, and during the COVID-19 pandemic from selected health facilities in Amhara region, Ethiopia, from July 7, 2019 to March 8, 2020, and from March 9, 2020 to July 6, 2020. This figure appears in color at www.ajtmh.org.

The mean monthly institutional delivery was 1,133 before the pandemic and in the pandemic months it has increased by 7.5% to 1,226 (Figure 4). The institutional delivery did not vary significantly between pre COVID-19 and during COVID-19.The average institutional delivery at primary healthcare facilities significantly increased during the COVID-19 period compared with pre-COVID-19 period (*P* = 0.03).

### Antenatal care follow-up.

**Figure 5.**
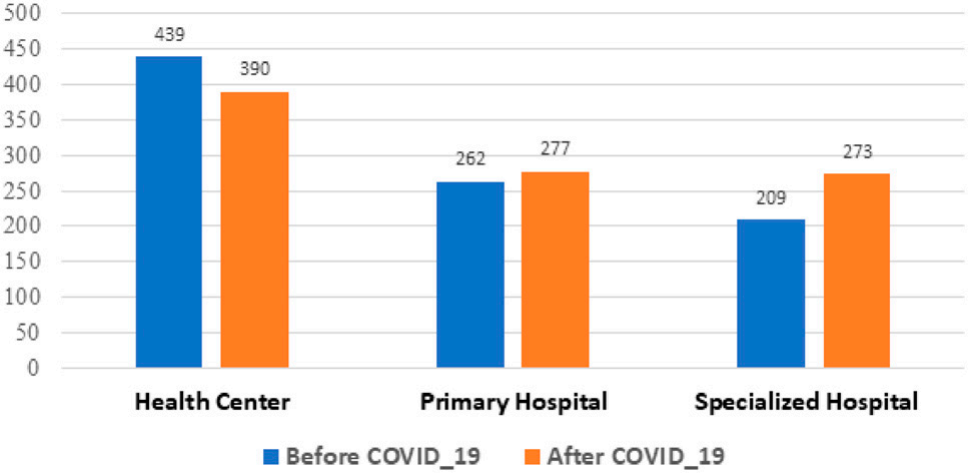
Average Antenatal care follow-up during pre-COVID-19, and during the COVID-19 pandemic period from selected health facilities in Amhara region, Ethiopia, from July 7, 2019 to March 9, 2020, and from March 13, 2020 to July 6, 2020. This figure appears in color at www.ajtmh.org.

The monthly mean of antenatal care (ANC) follow-up in pre-COVID-19 months was 910 and the pandemic period 941. The lowest number of first ANC visitors to health facility was seen in April (572) with about 38% decrease from the fiscal year mean with a rebound in May and June by 114 and 128% respectively (Figure 5). The ANC visit did not vary significantly between pre COVID-19 and during COVID-19.

**Figure 6.**
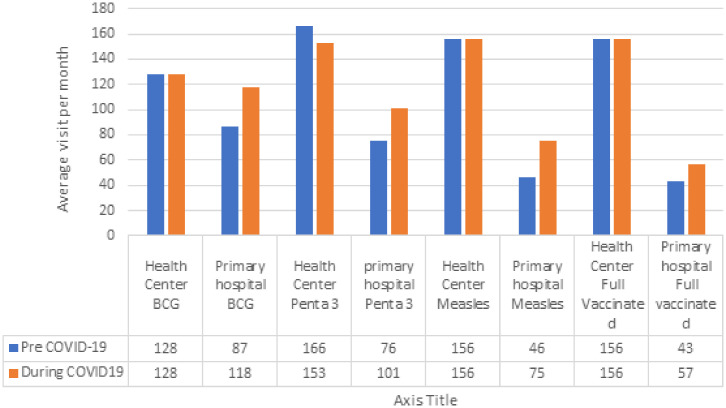
Average monthly visits for vaccinations during pre-COVID-19, and during the COVID-19 pandemic period from selected health facilities in Amhara region, Ethiopia, from July 7, 2019 to March 8, 2020, and from March 9, 2020 to July 6, 2020. This figure appears in color at www.ajtmh.org.

**Figure 7.**
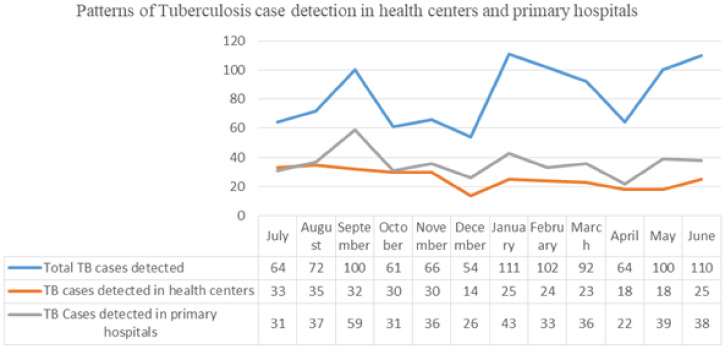
Patterns of new TB case detection during pre-COVID-19, and during the COVID-19 pandemic period from selected health facilities in Amhara region, Ethiopia, from July 7, 2019 to March 8, 2020, and from March 9, 2020 to July 6, 2020. This figure appears in color at www.ajtmh.org.

### Vaccination program.

The mean monthly number of children that received full immunization pre-COVID-19 and during the pandemic period was 468 and 469, respectively.

### Chronic disease services uptake.

In the pre-COVID-19 period, the monthly average diabetic patient visit was 1,078 and during the pandemic it dropped significantly by 31% to 745 (*P* = 0.001). Yet, the monthly average hypertension patients visiting health facilities dropped with 12% not significantly (*P* = 0.2) from 828 to 728, respectively.

### HIV care and treatment and TB case detection.

The mean number of HIV/AIDS patients who visited health facilities for drug refill and clinical evaluation on a monthly basis during the pre-COVID-19 period was 720 with no significant difference during the COVID-19 period (726). Yet, the mean number of newly diagnosed HIV/AIDS patients dropped by 43% during the COVID-19 period time compared with the pre-COVID-19 period.

The monthly average new TB case detection rate in the health centers and primary hospital was 93 before the lockdown and during the pandemic time, it decreased by only 4% to 89.

## DISCUSSION

Here we quantified the effect of the COVID-19 pandemic on the essential health services and the recovery of some health services. Patient flow for most essential health services (adult and pediatric outpatient visits, childhood immunization, surgical and medical emergency, chronic noncommunicable diseases) declined after the first COVID-19 case notification mid-March in Ethiopia. The lowest patients flow was documented in the subsequent month, April 2020. After this first decline, there was rapid recovery in patient flow for almost all essential health services (neonatal and child care, maternal health, chronic noncommunicable diseases, TB, and HIV).

Evidence from the 2013 to 2016 Ebola virus outbreak in western African countries indicated the negative, indirect effects of the epidemic on essential health services.^10^^,^^21^ Our study confirms an initial decline in the access of essential health services in the beginning of the COVID-19 pandemic (April 2020). The initial rigorous preventive measures including restriction of public transport and the public fear of contracting the disease may have contributed to the decreased client/patient flow. This may have directly or indirectly increased mortality and morbidities from treatable and preventable conditions. Yet, the disruption did not last long and in months of May and June the patient flow rebounded back to the prepandemic level. A study by Abdela et al., in one hospital in Ethiopia on essential healthcare visits, 4 weeks before and 4 weeks after the implementation of preventive measures concluded the decline of all essential healthcare services during COVID-19 preventive measure.^14^ The main limitation of the study was the short observation period that only included the first month of the pandemic coinciding with the most strict lockdown period so far.

The CDC and other reports have documented a marked decrease in vaccinations and primary prevention services during the pandemic.^21^^–^^23^ Here we found that the average childhood immunization in April decreased by 11% from the annual averages, 4 months later, the average childhood immunization was higher than the prepandemic period by about 11%. Also, there is no difference in the monthly average full immunizations before the pandemic (468) and after the pandemic (469), which is reassuring for the continuity of the health system.

In April 2020, Riley et al., used a mathematical model to forecast that “a 10% proportional decline in use of short- and long-acting contraceptive methods in low- and middle- income countries (LMICs) due to reduced access to healthcare services may lead to an additional 49 million women with an unmet need for modern contraceptives and an additional 15 million unintended pregnancies over the course of a year.” They also predicted that around a 10% decrease in maternal and new born care could result in catastrophic implications on the health of mothers and new born.^24^ In our observation, the pandemic did not show major effect on the number of institutional delivery and family planning services.

In June 2020, the WHO report on “rapid assessment of services delivery for non-communicable disease” stated that in countries where community transmission of COVID-19 is confirmed there is 66% and 64% of disruption services for hypertension and diabetes management, respectively.^25^ In our study on the monthly follow-up visit, there was only 18% and 31% declines in both hypertension and diabetes services, respectively.

There were concerns that countries with high burdens of TB and HIV/AIDS might be have difficulties in maintaining continuity of (quality) care to their patients, because of lockdowns restricting clinic access and the redeployment of financial and human resources away from HIV/AIDS and TB.^26^^,^^27^ Mohammed et al., reported that the new case detection of TB from one of the regions in Ethiopia from April to June (during the pandemic) has decreased about three times from the previous reporting periods. The number of new TB detection and HIV clinic follow-up was not affected by the pandemic in our study. However, the number of new HIV diagnosis decreased significantly during the pandemic period.

The MOH observed the worrisome declines in health services early in April. In collaboration with the regional health bureaus, the MOH circulated a directive for sustaining essential health service during the pandemic.^27^ In addition, in April 24, the MOH after giving a press release on the importance of maintaining essential health services, assigned a team of experts and decision makers to monitor the continuum of essential health services.^28^ We think that the reasons for the sustained and recovery of the essential health service are two factors. Firstly, the swift response by the MOH and regional health bureaus and secondly, the slow progression of the pandemic which never overwhelmed the health system.^12^ Apart from the essential health system, the authors recommend a further study to explore the full effect of the pandemic in the disease prevention programs, including the health extension program.

A strength of the study is its multicenter design involving different tiers of health facilities, therefore, it is representative of the Ethiopian healthcare units. Second, the study has looked at the data for an extensive whole fiscal year documenting the service of 8 months pre-COVID-19 period and 4 months during COVID-19 period. And third, we adhered to the STROBE guidelines (Strengthening the Reporting of Observational Studies in Epidemiology).^29^

The limitation of the study is that the study population did not include frontline health workers (health post) which are responsible for most of the preventive health services (family planning and immunization). Ideally, the pandemic timeframe of the periods (esp. during COVID-19 period) should be extended, to assess the effects later on during the COVID-19 pandemic. Additionally, comparison with the same months of previous years would account for the seasonality of some of the primary healthcare demands. But the 1-year data has also shown there is no significant variation in annual average patient flow, which is reassuring for the influence of seasonality.

Given that the pandemic is ongoing, such findings are of high interest to clinicians and policy makers in their effort to improve the resilience of health systems and hospitals during pandemics or similar disruptive events, including extreme climatic events. In conclusion, the study highlights the need for a strong program to assure essential healthcare components, while preventive procedures to mitigate a pandemic are imposed. The effect of public restrictions on essential care systems should be considered.
